# Supplementary material for: Oxygen transport in nanoporous SiN membrane compared to PDMS and polypropylene for microfluidic ECMO
Source: Biomed Microdevices. 2025 May 28;27(2):22. doi: 10.1007/s10544-025-00750-5 (PMC12119709; doi:10.1007/s10544-025-00750-5)
Supplement: Supplementary file 1 — Supplementary Material 1 [file 10544_2025_750_MOESM1_ESM.docx]

Supplementary figure


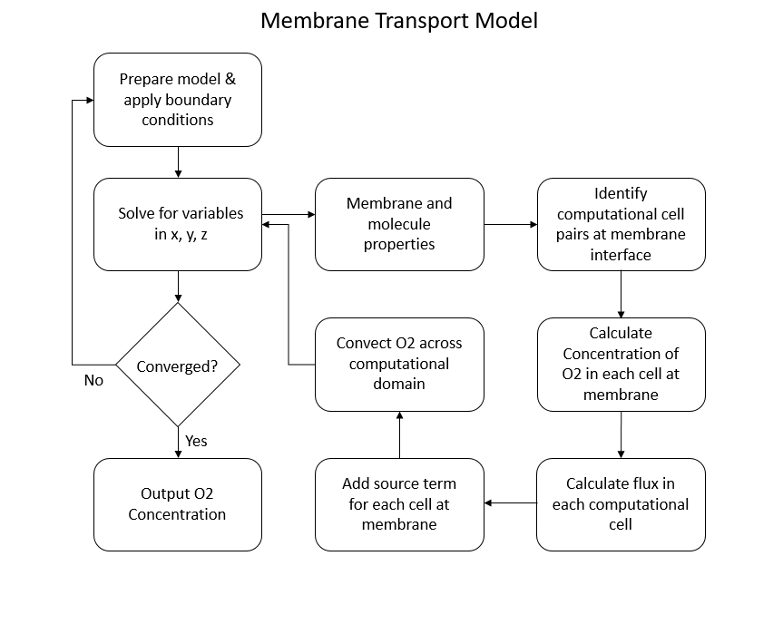


**Fig. S1** Flow chart showing the UDF process for oxygen transport across the membrane


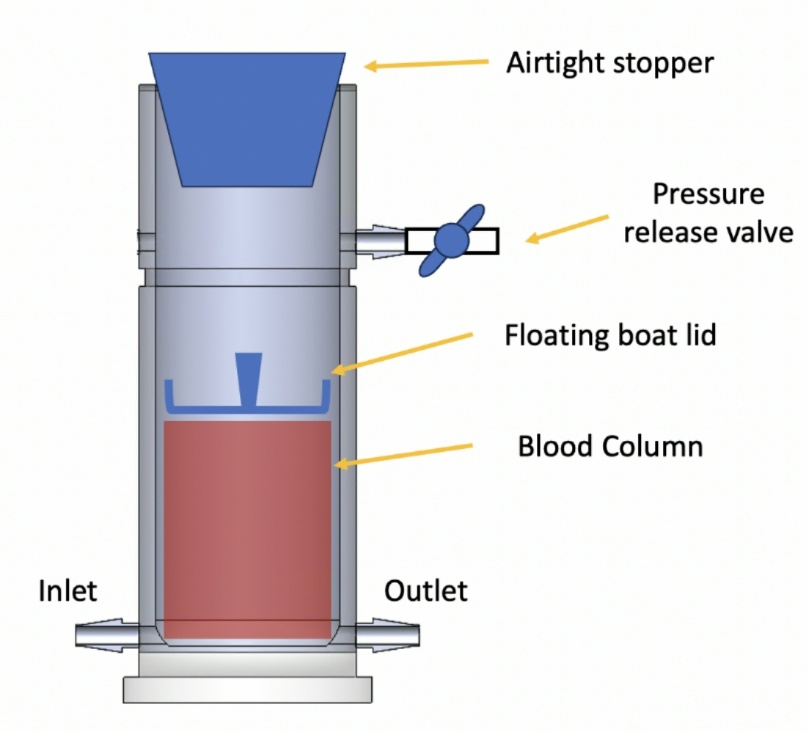


**Fig. S2** “floating boat lid” blood reservoir


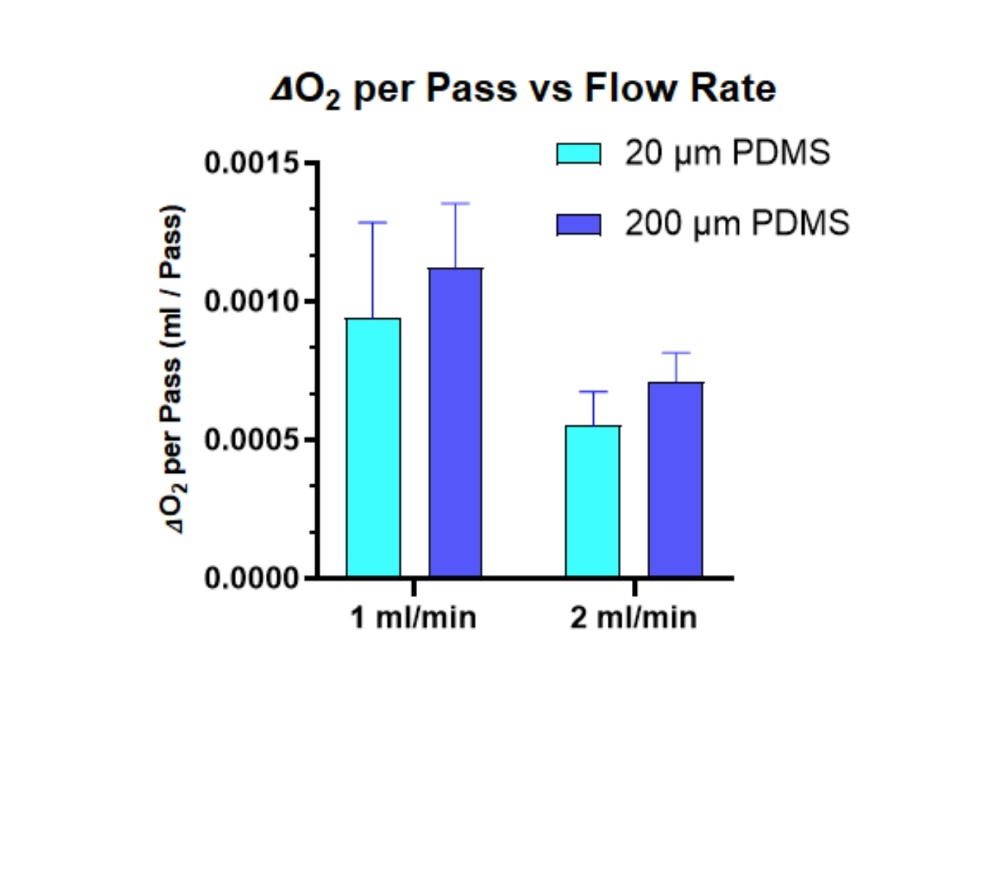


**Fig. S3** ∆O_2_ for water-water transport using PDMS membranes of 20 µm and 200 µm thickness
